# Accuracy of Delta Capnography for the Prediction of Pulmonary Vein Occlusion During Cryoablation for Atrial Fibrillation

**DOI:** 10.19102/icrm.2022.130302

**Published:** 2022-03-15

**Authors:** Juan Fernando Agudelo-Uribe, Juan David Ramirez-Barrera, Juan Alberto Espinal-Montoya, Andres Felipe Miranda-Arboleda, Gloria Saenz-Jaramillo, Marcela Paola Tobon-Upegui, Eduardo Castellanos-Martinez

**Affiliations:** ^1^Interventional Cardiology Department, CardioVID Clinic, Medellin, Colombia; ^2^Cardiovascular Surgery Department, CardioVID Clinic, Medellin, Colombia; ^3^Cardiology Department, CardioVID Clinic, Medellin, Colombia; ^4^Research Department, CardioVID Clinic, Medellin, Colombia; ^5^Cardiac Electrophysiology Unit, Monteprincipe University Hospital, Madrid, Spain

**Keywords:** Atrial fibrillation, capnography, cryoballoon, diagnostic test, pulmonary vein isolation

## Abstract

The purpose of this study was to quantify the relationship between a drop in end-tidal carbon dioxide (etCO_2_) and occlusion of pulmonary veins (PVs) to find a delta etCO_2_ (ΔetCO_2_) able to predict occlusion during PV isolation (PVI) by cryoballoon. We designed a prospective registry. Paroxysmal atrial fibrillation patients who underwent cryoballoon PVI were included. Capnography was performed. Occlusion was tested by injection. A comparison between ΔetCO_2_ and occlusion was performed. Eighteen subjects (138 injections) were included. A drop of >3.5 mmHg predicted occlusion of the PV (sensitivity, 80%; specificity, 86.7%). A ΔetCO_2_ of ≥3.5 mmHg during inflation of the cryoballoon in each PV directly correlates with PV balloon occlusion.

## Introduction

Atrial fibrillation (AF) is the most common sustained arrhythmia in humans. Despite good progress in its management, it remains one of the major causes of stroke, heart failure, sudden death, and dementia.^1^ Catheter ablation of AF has emerged as a common therapy to prevent recurrent AF since the initial description of triggers in the pulmonary veins (PVs) that initiate episodes of paroxysmal AF.^2^ This is achieved through isolation of the PVs, which is effective in restoring and maintaining sinus rhythm in patients with symptomatic paroxysmal, persistent, and probably long-standing persistent AF, in general, as a second-line treatment after failure or intolerance of anti-arrhythmic drugs.^1^

PV isolation (PVI) is the primary target of ablation.^1^ This is most commonly achieved by radiofrequency current application in a point-by-point mode, leading to cellular necrosis by tissue heating. An alternative method is the use of cryogenic energy applied with a balloon in a single-shot mode, which leads to necrosis by freezing the tissue. Radiofrequency ablation for AF requires only limited use of fluoroscopy and no use of contrast media because catheter guidance is achieved with the use of an electroanatomic mapping system, but this approach demands extensive training.^3^ In contrast, cryoablation for AF requires more extensive fluoroscopic guidance and the use of iodinated contrast to position the balloon catheter, but it creates a circular lesion around the PV in a relatively simple manner.^4^

Kuck et al.^4^ demonstrated that the cryoballoon ablation technique was non-inferior to radiofrequency ablation with regard to the primary efficacy endpoint (defined as the time to the first documented recurrence of AF, atrial flutter or tachycardia, prescription of anti-arrhythmic medicine, or re-ablation). There was no difference in the primary safety endpoint between the groups.

Applying a cryoballoon for PVI requires fluoroscopic guidance to navigate the balloon catheter to the left atrium. When occlusive circumferential contact is established at the PV ostium, the cryoballoon leads to durable PVI.^5^ PV occlusion is verified in a standard method by injecting contrast through the cryoballoon when it is insufflated into the vein, followed by observing the retention of the contrast agent in the distal PV branches by fluoroscopy.^6^ Other methods for PV occlusion testing include PV pressure waveform monitoring^7^ and intracardiac echocardiography (ICE) examination under Doppler flow display.^8^

When examining the long-term efficacy of a cryoballoon procedure, there are 3 important acute balloon-related biophysical parameters to consider. First, it is critical to ensure complete PV occlusion between the balloon and the antral PV surface.^9^ Second, during freezing, the time to acute intra-ablation PVI (time to isolation [TTI]) as recorded by an entrance and/or exit block test with a dedicated inner lumen circular diagnostic catheter is the most important parameter.^9^ However, not all PVs can be monitored for TTI because of the short (or missing) muscular sleeve extension into the PV.^9,10^ Finally, after delivering the freezing energy, the interval thaw time between the cryoballoon and the PV is another hallmark of an effective cryoablation procedure, with longer thaw durations favoring a more durable lesion as a consequence of proper balloon-to-tissue contact.^11^

Any technique able to predict the occlusion of the PV without the use of contrast media and fluoroscopy would be advantageous not only in terms of efficacy but also in terms of less radiation and contrast agent applied. If this can be achieved, the cryoballoon technique can be considered suitable for patients with renal failure. In addition, another measure of efficacy can potentially increase the reliability of the procedure.

Capnography determines the concentration of carbon dioxide (CO_2_) in patient-exhaled gases by side-stream gas sampling measured by infrared technology to detect CO_2_. It is part of standard monitoring during general anesthesia,^12^ and capnography is utilized to monitor systemic, pulmonary, and coronary blood flow.^13^ During capnography monitoring, cardiac output and end-tidal CO_2_ (etCO_2_) have a logarithmic relationship.^14^ In consequence, etCO_2_ is a sensitive indicator of pulmonary blood flow.^15^

**Figure 1:**
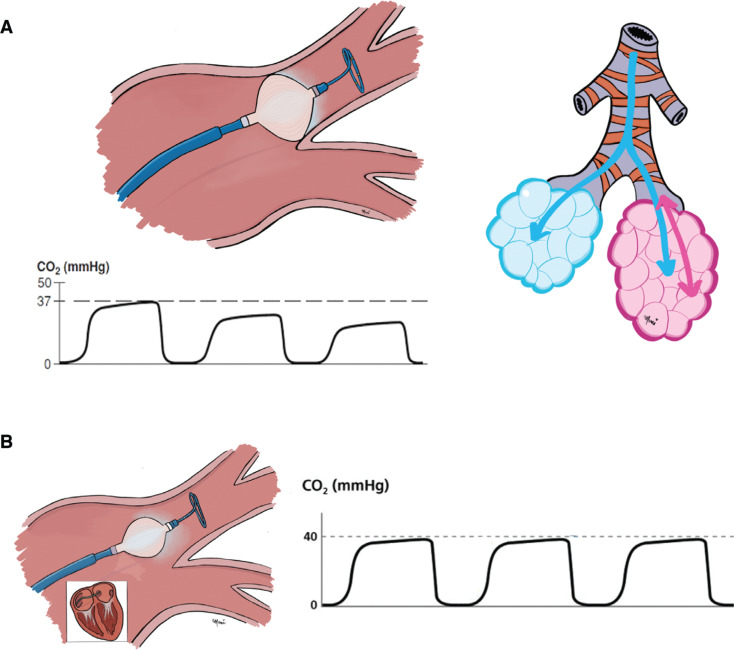
Physiological phenomena observed during inflation of cryoballoon. **(A)** During inflation of cryoballoon, regional pulmonary flow stops, generating a dead space. As a consequence, there is no gas interchange and drop in exhaled tidal CO_2_. **(B)** In contrast, if the cryoballoon does not occlude the PV, the capnography value remains stable.

The relationship between etCO_2_ and PV blood flow has been used as a potential marker for the ventilation– perfusion mismatch created by the occlusion of the PV by the cryoballoon.^16^ In fact, the use of capnography and the resulting change in etCO_2_ have been described previously as indicators of occlusion of PVI and long-term PVI efficacy^17^ **(Figure 1)**.

The main objective of this study was to quantify the relationship between the drop in etCO_2_ and the occlusion of the PV verified by the injection of contrast media in order to identify a “delta etCO_2_” (ΔetCO_2_) value able to predict occlusion of the PV.

## Methods

### Study design

We designed a prospective single-center registry in which all patients who underwent PVI using cryoballoon ablation in a cardiovascular-dedicated center were potential candidates for inclusion. The information of 18 patients treated between October 2019 and March 2020 was recorded consecutively in a pre-determined database added to by the researchers during every procedure. Patients assessed in our outpatient clinic, during admission because of AF or other cardiovascular conditions, or patients referred from other institutions, were considered to be enrolled in an “all-comers” way if they met the inclusion criteria of an age of >18 years, a diagnosis of paroxysmal AF despite ≥1 anti-arrhythmic drug, and suitability for a cryoballoon ablation procedure. Patients were excluded in cases of permanent or persistent AF, a severely dilated atria, non-favorable PV anatomy (in particular, a common antrum of the PVs), an intracavitary thrombus, mechanical valves, pregnancy, lack of patient information, or severe comorbidities. Capnography and etCO_2_ were performed for all patients as part of the standardized monitoring protocol for patients undergoing a cardiovascular intervention who required general anesthesia in our institution. All patients gave informed consent before the cryoablation procedure, and the confidentiality of all patients was protected. According to the institutional protocol, risk-free studies do not require approval by an ethics committee.

### Cryoballoon ablation stepwise approach

All cryoablation PVI procedures were performed using a second-generation 28-mm Arctic Front Advance^®^ cryo-catheter (Medtronic, Minneapolis, MN, USA). Pre-procedural work-up included contrast chest computed tomography (CT) imaging to evaluate the PVs and left atrium anatomy. A transesophageal echocardiogram was obtained ≥24 hours before the procedure to rule out intracardiac thrombi.

In patients with an indication for permanent anticoagulation either with direct oral anticoagulants or warfarin, the procedure was performed with a previous interruption of anticoagulant therapy 24–48 hours before the procedure or when the international normalized ratio ranged from 2–3.

All procedures were performed under general anesthesia. Vascular punctures were performed using ultrasound guidance. Vein accesses included the right femoral vein for 6-French (Fr) and 8-Fr sheaths, the left femoral vein with an 11-Fr sheath for ICE (Viewflex^®^; Abbott, Chicago, IL, USA), and right jugular vein access with a 7-Fr sheath for a decapolar (Inquiry^®^; Abbott) or duo-decapolar (Livewire^®^; Abbott) catheter placed in the coronary sinus. Immediately after the venous punctures, the patient was anticoagulated with sodium heparin at an initial bolus of 100 IU/kg with an activated clotting time target of 200–350 seconds during the procedure.

Through the 8-Fr sheath, a 0.032-in guidewire was advanced, over which a transseptal introducer sheath (Swartz^®^ Braided LAMP 90^®^; Abbott) was advanced. Using a 71-cm transseptal needle (BRK^®^; Abbott) and under fluoroscopic and direct ICE vision, transseptal catheterization was performed. Afterward, the 0.032-in guidewire was exchanged for a 260-cm-long high-support coated hydrophilic guidewire (Glidewire Advantage^®^ 0.035; Terumo Medical Corporation, Somerset, NJ, USA), and a FlexCath Advance™ 15-F steerable sheath (Medtronic) was then placed in the left atrium.

Guided by ICE and fluoroscopy and using CT imaging of the left atrium as an anatomic reference, the cryoballoon and the Achieve^®^ circular mapping catheter (Medtronic) were advanced into the left atrium and subsequently into every PV ostium. The Achieve^®^ catheter was advanced into every vein and the cryoballoon (Arctic Front Advance^®^; Medtronic) was insufflated over it, with iodinated contrast subsequently injected through the distal tip of the catheter to test the complete sealing of the PV before proceeding with cryoablation during 240 seconds. In cases of incomplete sealing, a new position of the Achieve^®^ catheter in another vein branch was adopted and iodinated contrast was injected again until an acceptable seal between the vein and cryoballoon was achieved. An absence or dissociation of PV potentials was confirmed. In cases with persistent non-dissociated potentials, a second lesion was created. Additional lesions were performed at the discretion of the main operator. During the right PVI, simultaneous phrenic nerve stimulation was carried out to detect its lesion during ablation. Simultaneously, basal and minimal capnography values during each insufflation were recorded. On the right side, a record was taken before starting phrenic nerve stimulation.

### Test methods

#### Capnography (ΔetCO_2_)

Side-stream capnography is routinely performed in all patients who undergo major cardiovascular procedures under general anesthesia at our institution. Capnometry of etCO_2_ is measured in the exhaled gases using infrared light to generate a capnography curve that is used to supervise organ perfusion due to the direct proportional relationship between etCO_2_ and cardiac output. Likewise, etCO_2_ can be a marker of a ventilation–perfusion mismatch created during PV occlusion while the cryoballoon is inflated during cryoablation.

Orotracheal intubation was performed following the standard institutional protocol. A Datex-Ohmeda Aestiva 5^®^ anesthesia machine (Datex-Ohmeda, Madison, WI, USA) with a corresponding full-size patient monitor (etCO_2_ monitor included) was used in each procedure. Patients were ventilated under the assisted-controlled ventilation mode (either with volume or pressure) through endotracheal intubation. A tidal volume of 6–8 mL/kg was programed. The respiratory rate was adjusted to maintain an etCO_2_ of 28–38 mmHg. Hemodynamic variables (arterial blood pressure and heart rate) were intervened to keep them as stable as possible in order to avoid or minimize any variations in etCO_2_ (the goal of mean arterial pressure was 50–70 mmHg, and the heart rate was 60–90 bpm). If needed, a noradrenaline infusion or cardiac stimulation (through a decapolar or duo-decapolar catheter) was used. The etCO_2_ was registered in the pre-designed form before, during, and after each cryoablation session.

The capnography delta (ΔetCO_2_) value was calculated from the difference between the baseline capnography values in every patient and the minimum capnography during ablation for every application in each vein. To our knowledge, there are no reports in the literature that describe a cutoff of ΔetCO_2_ that correlates with vein occlusion guided by contrast media injection and fluoroscopy.

#### Reference standard

PV occlusion after insufflation of the cryoballoon is the standard practice assessed by the electrophysiologist as the main operator. During the procedure, once the cryoballoon and the Achieve^®^ catheter are placed in the left atrium, both are navigated to every PV ostium. Once inside each PV, the balloon is insufflated, and contrast media is injected through the tip of the catheter. If the vein is completely sealed, there will not be any contrast regurgitation to the left atrium visualized with fluoroscopy. The position of the cryoballoon relative to each vein is also supervised with the help of ICE. This is a qualitative and subjective method; however, there is no other standardized technique available to objectively determine this key endpoint. On the other hand, it is the method used by the pivotal clinical trial.^4^ Based on these parameters, the operator defines whether the PV is completely occluded and, afterward, cryoablation is started. Full sealing between the PV ostium and cryoballoon is a milestone during the procedure because this is directly related to its long-time effectiveness, the minimum temperature achieved, number of applications, iodinated contrast total volume, radiation dose, and total procedure time.

### Statistical analysis

Continuous data were summarized depending on a normal or non-normal distribution using mean with standard deviation (SD) values or median with interquartile range (IQR) values, while categorical variables were given as absolute numbers and percentages. Comparisons between groups were performed using Student’s *t*-test with statistical significance if the *P* value was <.05.

The main outcome was to determine a cutoff for ΔetCO_2_ that predicts PV occlusion compared to the standard of reference, which, in this study (and in general clinical practice), was the vein occlusion appreciation of the operator. The diagnostic accuracy of the values of ΔetCO_2_ was assessed during receiver operating curve (ROC) analysis, presented with an area under the ROC curve (AUC) with 95% confidence intervals (CIs). Additional diagnostic parameters, such as sensitivity, specificity, and likelihood ratio (LR), were also determined. Data were not imputed, and missing capnography values were excluded from the analysis. Sample size was not calculated as all patients who underwent cryoablation were included. Statistical analyses were completed in SPSS Statistics version 20 (IBM Corporation, Armonk, NY, USA).

## Results

### Study population

A total of 18 subjects with symptomatic paroxysmal AF were included. Eight of them had associated cavo-tricuspid isthmus–dependent flutter. According to the modified European Heart Rhythm Association classification,^1^ 55.6% had 2a (mild) symptoms. One hundred thirty-eight cryoablation applications were performed. Among these, 108 of them occluded the PV as confirmed by contrast media injection and fluoroscopy. The average age of the population was 51.5 years (SD, 11 years). Twelve patients (66.7%) were men. The main comorbidity found was dyslipidemia in 8 patients (44.4%). None of them had a history of coronary heart disease or previous cardiac surgery. Half of the patients were previously treated with apixaban (22.2%) or rivaroxaban (27.8%). Six had no previous anti-thrombotic management. Every patient was on an anti-arrhythmic, mainly metoprolol tartrate (61.1%). Their median left ventricular ejection fraction as documented by echocardiography was 60%. The left atrium area and volume average were 18.6 cm^2^ (SD, 3.89 cm^2^) and 26.5 cm^3^ (21–34 cm^3^), respectively. In 6 patients, an accessory vein was observed during the left atrial CT scan. One patient presented with cardiac tamponade requiring percutaneous drainage (this complication probably occurred during manipulation of quadripolar catheter for stimulation of the phrenic nerve). The other procedures were performed without any complications **(Table 1)**.

### Main findings

**Figure 2:**
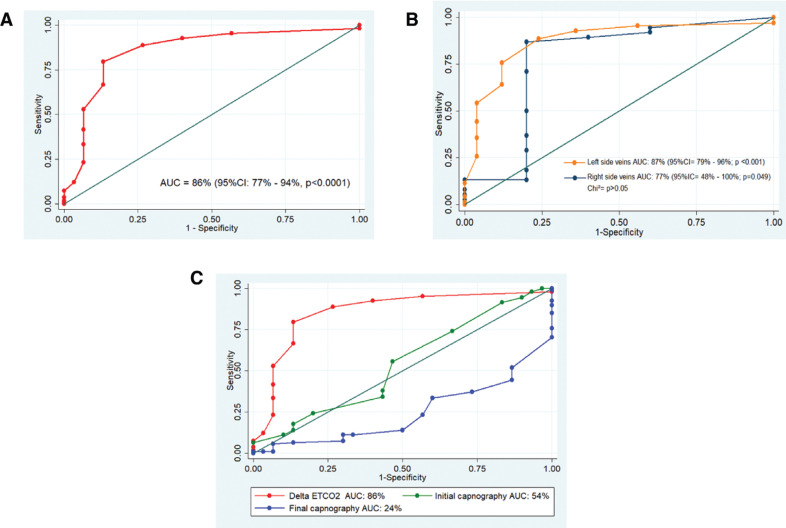
Area under the receiver operating characteristic (ROC) curve (AUC) analysis for of delta end-tidal CO_2_ (ΔetCO_2_) during cryoballoon, **(A)** overall ROC analysis, **(B)** left-side veins versus right-side veins, and **(C)** AUC analysis for initial and final capnography versus ΔetCO_2_. *Abbreviations:* AUC, area under the receiver operating characteristic curve; CI, confidence interval; ΔetCO_2_, delta end-tidal CO_2_; ROC, receiver operating curve._._

Baseline and minimal capnography measures are described in **Table 2**. Also, values classified by the side of the veins are shown. There were no significant differences in either baseline, minimal, or delta capnography when classified by right- or left-side veins (*P* > .05, Mann–Whitney *U* test). Considering both sides, the overall ROC analysis showed an AUC of 86% (95% CI, 77%–94%; *P* < .0001) **(Figure 2A)**. Further analysis proved that a ΔetCO_2_ of >3.5 mmHg had the best accuracy cutoff to predict PV occlusion during the cryoballoon ablation procedure, with a sensitivity of 80%, a specificity of 86.7%, a positive LR of 6, a positive predictive value of 96%, and a negative predictive value (NPV) of 54%.

Additional accuracy diagnostic parameters were assessed by every side vein to look for differences depending on the left or right side based on the differential cardiac output by side or the effect exerted by stimulation of the phrenic nerve **(Table 3)**. Left-side veins showed a better diagnostic performance with an AUC of 87% (95% CI, 79%–96%; *P* < .0001) **(Figure 2B)**. Detailed information about diagnostics for left-side veins with a ΔetCO_2_ cutoff of 3.5 is reported **(Table 3)**. Right-side veins had a lower AUC compared to the left-side veins at 77% (95% CI, 48%–100%; *P* = .049) **(Figure 2B)**. However, the difference was not statistically significant (*P* > .05, chi-squared test). For right-side veins, the best cutoff was also established with a ΔetCO_2_ of 3.5 mmHg **(Table 3)**. An exploratory analysis using other capnographic variables, such as the initial capnography and final capnography values, was carried out. However, none of them showed an adequate diagnostic accuracy compared to ΔetCO_2_.

There were 6 patients with accessory PVs **(Table 1)**. The mean diameter was 11.87 mm. In those cases, we attempted to isolate the PV with the same balloon. The response in terms of the drop in capnography was attenuated as a consequence of less flow interrupted. However, given that we did not analyze the drop in capnography for each vein, it is not possible to precisely know the effect of occlusion for each accessory vein.

## Discussion

The main finding of this exploratory diagnostic registry is the confirmation that, during a cryoballoon PVI procedure, a drop in the capnography value successfully predicted complete occlusion of the PVs. In fact, our data show that a drop of >3.5 mmHg predicts occlusion of the PV with a sensitivity of 80% and a specificity of 86.7%. There was no significant difference between the right- and left-side veins.

In 2015, Hoyt and Lim^16^ described and quantified the capnographic changes during cryoballoon PVI. They determined the utility of end-tidal CO_2_ as an indicator of PV occlusion during ablation. In their study, 80 PVs were acutely isolated with a cryoballoon. The sensitivity for PV occlusion was 90% for the right superior PV, 70% for the left superior PV, and 50% for both the right and left inferior PVs. These findings showed a lower sensitivity compared to ours. One possible explanation is the greater proportion of gas exchange occurring in the upper lobes during general anesthesia. Depending on the ventilatory technique chosen, the lower lobes may remain under-ventilated. They originally used this method as a primary tool in order to avoid or minimize contrast agent administration, in particular for patients with renal dysfunction or pre-diagnosed contrast agent allergies. They preferred to use this method as a complementary tool to assess the maintenance of the occlusion during PVI. This method was found to be very useful in obese patients in whom visualization of contrast media is difficult due to a reduction of fluoroscopy penetration across a larger body mass. Also, they mentioned the utility of this method during repositioning of the balloon for repeated ablation applications. Additionally, they demonstrated that there was no influence of the rhythm (AF or sinus rhythm) on this physiological finding.

In 2016, Sircy et al.^10^ performed a retrospective examination of observational data from 15 patients who underwent a cryoballoon PVI procedure. They reviewed and recorded the capnography readings during those procedures and the parallel cryoballoon catheter performance parameters (including balloon nadir temperature, freeze duration, and TTI) during each cryoablation shot. They found a direct correlation between nadir temperature and the capnography nadir of etCO_2_ (*P* = .0001 for the Pearson correlation). Interestingly, in their study, most etCO_2_ nadirs were typically achieved within 60 seconds of PV-to-balloon occlusion.

In 2018, Pickett et al.^17^ evaluated 30 subjects who underwent cryoballoon PVI for a drug-refractory symptomatic paroxysmal AF. They monitored and used etCO_2_ to evaluate the occlusion of each PV. Subjects were monitored during long-term follow-up for the recurrence of AF. Strikingly, they excluded patients with a clinical history of diabetes or dysautonomia (they presumed that these conditions could alter the baseline hemodynamics and efficacy of the response to catheter ablation therapy). They used the same protocol for etCO_2_ sampling previously described by Hoyt and Lim.^16^ The primary efficacy endpoint was the 1-year outcome of the cryoballoon ablation procedure. The secondary endpoint was to determine whether any etCO_2_ level could be used to differentiate between subjects who had recurrent AF and subjects who maintained a normal sinus rhythm. As in our study, they calculated the ΔetCO_2_ (defined by subtracting the nadir etCO_2_ from the basal etCO_2_) at each PV measured before each cryoablation. They followed all patients for 1 year after the index cryoablation. Of the 30 subjects, 5 had a recurrence of atrial arrhythmia (>30 seconds), meaning a long-term success rate of 83.3%. The main finding of this study was a significant difference in ΔetCO_2_ between the sinus rhythm and recurrent AF patients (6.8 ± 5.0 mmHg in the sinus rhythm group vs. 4.1 ± 4.2 mmHg in the recurrent AF group, *P* < .001). However, a definitive “cutoff” value useful for predicting the persistence of a sinus rhythm at 1 year was not found due to the large overlap in values between the groups. Another interesting finding of their study was that failures to achieve a change in etCO_2_ in 60 seconds could be used with other parameters to terminate an unsuccessful freeze attempt.

The most important limitation of our registry is that, in some cases, the main operator, who was responsible for determining the PV occlusion by the conventional method (the standard for comparison), was not blinded to the value of etCO_2._ Therefore, there is a possibility of bias toward occlusion determination by the standard method when a significant drop in etCO_2_ was observed. Another important limitation is the low NPV obtained. It must be recognized that this is an exploratory registry with few patients. Its primary purpose was to test a concept and estimate a cutoff point for ΔetCO_2_. A larger study is needed to get more accurate specificity and sensibility values. In this protocol, PV tomography was used to characterize the anatomy of each PV. As a consequence, use of a 3-dimensional mapping system was not necessary. Finally, every procedure was performed under general anesthesia to favor the patient’s comfort, allow the use of an esophageal thermometer, and stabilize ventilatory parameters. The general anesthesia protocol was standardized to eliminate variations in the respiratory pattern as a potential cause of variations in capnography measurements. We do not know whether carrying out the procedure under sedation allows us to appreciate the changes in capnography. To avoid phrenic stimulation from affecting ΔetCO_2_, each right PV was occluded, and we waited for a drop in capnography to make a measurement. Then, cooling was started with simultaneous phrenic stimulation. Practicing the procedure under deep sedation could be an alternative to explore in the near future to verify if the same changes in capnography can be measured.

To the best of our knowledge, this is the first prospective registry that has achieved an accurate determination of a capnography cutoff value (ΔetCO_2_) able to predict the occlusion of each PV with an acceptable level of sensitivity and specificity for each PV, with independence from the PV side. The main advantage of this tool was the reductions in contrast media and fluoroscopy use to determine PV occlusion. In contrast with other techniques (such as intracardiac echo), this measure can be performed in each PV no matter whether there are anatomic or positional variations. Another advantage of this technique is that no additional devices are needed (such as a direct measure of PV pressure during the occlusion), and it can be practiced routinely without increasing the risks for patients or the cost.

## Conclusions

In conclusion, a drop in etCO_2_ (ΔetCO_2_) of ≥3.5 mmHg in capnography during inflation of the cryoballoon in each of the PVs directly correlates with PV balloon occlusion. The measurement of ΔetCO_2_ constitutes a very simple, reliable, and universally available method for monitoring the PV occlusion during PVI by cryoballoon that allows for the use of less contrast media and fluoroscopy.

A prospective study in which the main operator is blinded to the capnography results is necessary in order to avoid the previously described risk of bias. Additionally, it would be very useful to know whether there is a direct correlation between ΔetCO_2_ and freedom from atrial arrhythmia at 1-year follow-up.

## Figures and Tables

**Table 1:** Baseline Patient Characteristics

Baseline Characteristics	Total Cohort n = 18
General	
Age, years	51.7 (11.01)
Male	12 (66.7%)
Associated cavotricuspid-dependent isthmus flutter	8 (44.4%)
Comorbidities	
None	6 (33.3%)
Dyslipidemia	8 (44.4%)
Hypertension	7 (38.9%)
Obstructive sleep apnea–hypopnea syndrome	4 (22.2%)
Type 2 diabetes mellitus	1 (5.6%)
Stroke	1 (5.6%)
Congestive heart failure	1 (5.6%)
Chronic kidney disease	1 (5.6%)
Previous antithrombotic management	
None	6 (33.3%)
Rivaroxaban	5 (27.8%)
Apixaban	4 (22.2%)
Acetyl-salicylic acid	2 (11.1%)
Previous anti-arrhythmic management	
Metoprolol tartrate	11 (61.1%)
Metoprolol succinate	4 (22.2%)
Propafenone	4 (22.2%)
Amiodarone	3 (16.7%)
Bisoprolol	1 (5.6%)
Carvedilol	1 (5.6%)
Additional management	
None	10 (55.6%)
Calcium antagonist	1 (5.6%)
ACEI	2 (11.1%)
ARA2	5 (27.8%)
Diuretic	2 (11.1%)
Statin	4 (22.2%)
Severity of symptoms (modified EHRA scale)	
Mild (2a)	10 (55.6%)
Moderate (2b)	7 (38.9%)
Severe (3)	1 (5.6%)
Echocardiographic findings	
Ejection fraction (%)	60
Left atrial area (cm^2^)	18.6 (SD: 3.89)
Left atrial volume (cm^3^)	26.5 (21–34)
Left atrial CT scan findings	
Normal configuration (four veins, separated antrum)	12 (66.7%)
Accessory vein (five veins, separated antrum)	6 (33.3%)
Left superior pulmonary vein diameter	18.8 (SD: 4.10)
Left inferior pulmonary vein diameter	16.73 (SD: 2.91)
Right superior pulmonary vein diameter	17.13 (SD: 2.35)
Right inferior pulmonary vein diameter	17 (15–19)
Accessory pulmonary vein diameter	11.87 (SD: 4.21)

*Abbreviations:* ACEI, angiotensin-converting enzyme inhibitor; ARA2, angiotensin receptor type 2 antagonist; CT, computed tomography; EHRA, European Heart Rhythm Association. Continuous variables are given as mean and standard deviation (SD) values. Count variables are given as number and percentage values.

**Table 2:** Baseline and Minimal Capnography Values

	Median of Both Sides (IQR)	Median of Right-side Veins (IQR)	Median of Left-side Veins (IQR)	*P* value of Right vs. Left Side^a^
Baseline capnography (mmHg)	34 (4)	34 (6)	33.5 (4)	.41
Minimal capnography (mmHg)	29 (5)	27 (6)	29 (5)	.29
Delta capnography—ΔetCO_2_ (baseline – minimal value)	5 (6)	5 (4)	4.5 (6)	.138

*Abbreviations:* ΔetCO_2_, delta end-tidal carbon dioxide, IQR, interquartile range.

^a^Mann–Whitney *U* test.

**Table 3:** Diagnostic Parameters for ΔetCO_2_ During Cryoballoon Pulmonary Vein Isolation for Atrial Fibrillation

	Cutoff	AUC (%) (95% CI)	Sensitivity (%)	Specificity (%)	PPV (%)	NPV (%)	LR (+)	*P* value
Both sides	3.5	86 (77–94)	80	86.7	96	54	6	<.0001
Left-side veins	3.5	87 (79–96)	76	88	95	56	6.3	<.0001
Right-side veins	3.5	77 (48–100)	87	80	97	44	4.35	.049

*Abbreviations:* ΔetCO_2_, delta end-tidal CO_2_; AUC, area under the receiver operating characteristic curve; CI, confidence interval; PPV, positive predictive value; NPV, negative predictive value; LR, likelihood ratio.
